# A companion to Part 2 of the World Checklist of Opiliones species (Arachnida): Laniatores – Samooidea, Zalmoxoidea and Grassatores incertae sedis

**DOI:** 10.3897/BDJ.3.e6663

**Published:** 2015-12-21

**Authors:** Adriano B. Kury, Abel Pérez-González

**Affiliations:** ‡ Museu Nacional, Universidade Federal do Rio de Janeiro, Rio de Janeiro, Brazil; § Museo Argentino de Ciencias Naturales "Bernardino Rivadavia" - CONICET, Buenos Aires, Argentina

**Keywords:** New genus, new familial assignments, new synonymies, genital morphology, Gonyleptoidea, Phalangodoidea, Brazil, Ecuador, Indonesia, Madagascar, Mozambique, Peru, Venezuela

## Abstract

**Background:**

A series of databases is being prepared to list the valid species of Opiliones worldwide. This paper containing nomenclatural acts is meant to accompany Part 2, which includes the members of the infraorder Grassatores of the superfamilies Samooidea and Zalmoxoidea plus the Grassatores currently not allocated to any family (*i.e.* Grassatores incertae sedis).

**New information:**

The following 32 taxonomic changes are proposed here:

(1-3) The Afrotropical genera *Hovanoceros* Lawrence, 1959, *Malgaceros* Lawrence, 1959 and *Tetebius* Roewer, 1949 (all currently in Samoidae) are all newly transferred to Biantidae.

(4-5) *Microminua soerenseni* Soares & Soares, 1954, from Brazil is newly transferred to *Tibangara* (Gonyleptoidea: Cryptogeobiidae), newly combined as *Tibangara soerenseni* new comb., new familial allocation for the species.

(6-7) The new genus *Llaguenia* Gen. nov is erected for the South American species *Zamora peruviana* Roewer, 1956, newly combined as *Llaguenia peruviana* new comb., and newly placed in Gonyleptoidea: Cranaidae (Prostygninae).

(8) *Bebedoura* Roewer, 1949, known from a single Brazilian species, is transferred from Tricommatinae to Grassatores incertae sedis.

(9) *Microconomma* Roewer, 1915, known from a single Cameroonian species, is transferred from Samoidae to Grassatores incertae sedis.

(10) *Stygnomimus* Roewer, 1927, with two Indomalayan species and hitherto included in the Stygnommatidae, is here formally considered Grassatores incertae sedis.

(11) *Bichito* González-Sponga, 1998, known from a single Venezuelan species, originally described in Phalangodidae: Phalangodinae, and currently in Grassatores incertae sedis is transferred to Samoidae.

(12) The Neotropical genus *Microminua* Sørensen, 1932, currently with two species, is newly transferred from Kimulidae to Samoidae.

(13-14) *Cornigera* González-Sponga, 1987 (currently in Samoidae), is newly considered a junior subjective synonym of *Microminua*, and its single species is combined under *Microminua* as *Microminua flava* (González-Sponga, 1987) new comb.

(15) *Niquitaia* González-Sponga, 1999 (originally in Phalangodidae: Phalangodinae, currently in Zalmoxidae), monotypic from Venezuela, is newly transferred to Samoidae.

(16) *Heteroscotolemon* Roewer, 1912 originally described in Phalangodidae: Phalangodinae, and currently in Grassatores incertae sedis is transferred to Zalmoxidae.

(17) While the Australasian genus *Zalmoxista* Roewer, 1949 is currently in Samoidae and some of its former species have been transferred to *Zalmoxis* Sørensen, 1886, *Zalmoxista americana* Roewer, 1952 from Peru, is here newly transferred to Zalmoxidae into *Minuides* Sørensen, 1932, forming the combination *Minuides americanus* (Roewer, 1952) new comb. (specific name inflected to match the masculine gender).

(18) *Neobabrius* Roewer, 1949 (currently in Phalangodidae), monotypic from Indonesia, is newly transferred to Zalmoxidae.

(19) While *Crosbyella* Roewer, 1927, belongs to Phalangodidae, *Crosbyella roraima* Goodnight & Goodnight, 1943 (originally Phalangodinae, but currently Zalmoxidae without generic assignment) is here transferred to *Soledadiella* González-Sponga, 1987, as *Soledadiella roraima* new comb. (Zalmoxoidea: Zalmoxidae).

(20) *Zalmoxissus* Roewer, 1949 is newly synonymized with *Zalmoxis* Sørensen, 1886 (Zalmoxidae).

(21) The original spelling *Zalmoxis sorenseni* Simon, 1892 is restored from the unjustified emendation *soerenseni*.

(22) The Neotropical genus *Phalangodella* Roewer, 1912 (originally in Phalangodidae: Tricommatinae, but currently in Grassatores *incertae sedis*) is newly transferred to Zalmoxoidea *incertae sedis* and (23-26) four other genera are **newly synonymized** with it: *Phalangodella* Roewer, 1912 = *Exlineia* Mello-Leitão, 1942 = *Langodinus* Mello-Leitão, 1949 = *Cochirapha* Roewer, 1949 = *Phalpuna* Roewer, 1949, generating the following **new combinations** (27-32): *Phalangodella fulvescens* (Mello-Leitão, 1943) new comb., *Phalangodella milagroi* (Mello-Leitão, 1942) new comb., *Phalangodella rhinoceros* (Mello-Leitão, 1945) new comb., *Phalangodella flavipes* (Mello-Leitão, 1949) new comb., *Phalangodella rugipes* (Roewer, 1949) new comb. and *Phalangodella urarmata* (Roewer, 1949) new comb.

## Introduction

This work is a companion to the 2nd data paper in a series containing a checklist of the valid species of harvestmen in the World: "World Checklist of Opiliones species (Arachnida). Part 2: Laniatores – Samooidea, Zalmoxoidea and Grassatores *incertae sedis*" (Kury et al. 2015). Herein, reasoning for taxonomic decisions is given associated with nomenclatural acts to clarify new taxonomic changes in ​ the list presented in that work.

Harvestmen taxonomy, founded in the classic and typological Roewerian system (*e.g.* Roewer 1912) has been criticized by several authors. Discarding of the hierarchical weighting of the typical Roewerian morphological diagnostic characters and the use of alternative sources of morphological and molecular information and the biogenetic framework allowed the appearance of a new distinct classification system that caused a revolution in Opiliones systematics.

We are currently in the middle of this systematic reorganization. Modern approaches are very different to the classical ones, and we now typically have a critical amount of new information about the arrangement of the major Opiliones lineages that is highly incongruent with the Roewerian system. But there is a huge impediment – a considerable fraction of harvestmen species are known only from their original and often insufficient descriptions, and their names remain tied to an obsolete taxonomic system. Huge efforts were made by modern opilionologists (*e.g.* Kury 2003) to utilize the poor information available for an enormous amount of taxa, and to accommodate them into a more natural system. However, this is not enough and many harvestmen still remain as *incertae sedis* in several taxonomical categories.

For that reason it is not rare for newly written compilations in Opiliones, such as catalogues, checklists, *etc.*, to typically include several taxonomic and nomenclatural changes (*e.g.* Kury 2003). With critical reading of the published bibliography (mainly the old works), re-examination of each type specimen, examination of male genital morphology drawings, sequencing of DNA, *etc.* comes important hints to make some of the immeasurable changes needed in this new harvestmen taxonomic system. The current work deals with one of the most challenging and frustrating segments of Opiliones systematics,the superfamilies Samooidea and Zalmoxoidea, which have been extensively worked on in recent years including huge changes in their taxonomy and systematics (*e.g.* Kury 2003, Sharma and Giribet 2011, Sharma et al. 2011a, Pérez-González and Kury 2007b, Sharma et al. 2011b). Increasing amounts of phylogenetic evidence (*e.g.* Sharma and Giribet 2011) support both lineages as natural, corroborating their excision from the nonsense Roewerian Phalangodidae – one of the most resilient bastions of the ever crumbling Roewerian system. Likewise, the Samooidea and Zalmoxoidea include the highest number of *incertae sedis* taxa which still often challenge the work of the harvestmen taxonomists today.

## Material and methods

The large collections of our own musems, MNRJ (Museu Nacional/ UFRJ, Rio de Janeiro) and MACN (Museo Argentino de Ciencias Naturales "Bernardino Rivadavia", Buenos Aires), have been complemented along the years by several visits to major repositories in the world. Re-study of type specimens and search for homologies in genital morphology associated with a cladistic framework allowed us to dismantle Roewer's system. The advent of the internet and wide digitalization of literature allowed access to countless rare works, allowing spelling checks, and solidly anchoring checklists.

Optical photograph images were taken along the years with a variety of hardware. Most recent photos have been integrated with the stacker software CombineZP Suite (Hadley 2009) based on preliminary images at different focal planes.

Male genitalia preparation follows Acosta et al. (2007), and were temporarily mounted in glycerol. Penis were drawn using a camera lucida attached to different kinds of compound microscopes. Genitalia ink drawings were digitalized and vectorized using Corel Draw X7. All figures were edited using Photoshop CS5 and Corel Draw X7 softwares.

**Abbreviations: AK** = Adriano Kury reference number; **AMNH** = American Museum of Natural History, New York, USA; **FNMH** = Field Natural History Museum, Chicago, USA; **MCNC =** Museo de Ciencias Naturales de Caracas, Caracas, Venezuela; **MNHN** = Muséum national d'Histoire naturelle, Paris, France; **MSNG =** Museo Civico di Storia Naturale "Giacomo Doria", Genoa, Italy; **MZSP** = Museu de Zoologia da Universidade de São Paulo, Brazil; **SMF** = Naturmuseum Senckenberg, Frankfurt am Main, Germany; **ZMUC =** Universitetets Zoologiske Museum Copenhagen, Denmark.

## Results

### Biantidae

There are two closely related genera from Madagascar and a third from Eastern Continental Africa which could all be closely related. They are here included in Biantidae.

***Hovanoceros* Lawrence, 1959, new familial allocation**

*Hovanoceros* Lawrence 1959: 71 [type species: *Hovanoceros bison* Lawrence, 1959, by original designation].

***Hovanoceros bison* Lawrence, 1959**

*Hovanoceros bison* Lawrence 1959: 71.

**Type data.** 2 **♂** syntypes (MNHN, not examined) MADAGASCAR, Ambodivoangi, Maroantsetra.

**Historical systematic background.** Lawrence (1959) described the monotypic *Hovanoceros* from Madagascar, including in it in the Phalangodidae: Samoinae, along with *Malgaceros* and *Anaceros*. Staręga (1992) included only *Anaceros* in Biantidae (probably because of the lack of a common ocularium), leaving the other two in Samoidae. There are a number of undescribed species currently under study.

**Figure 1.**
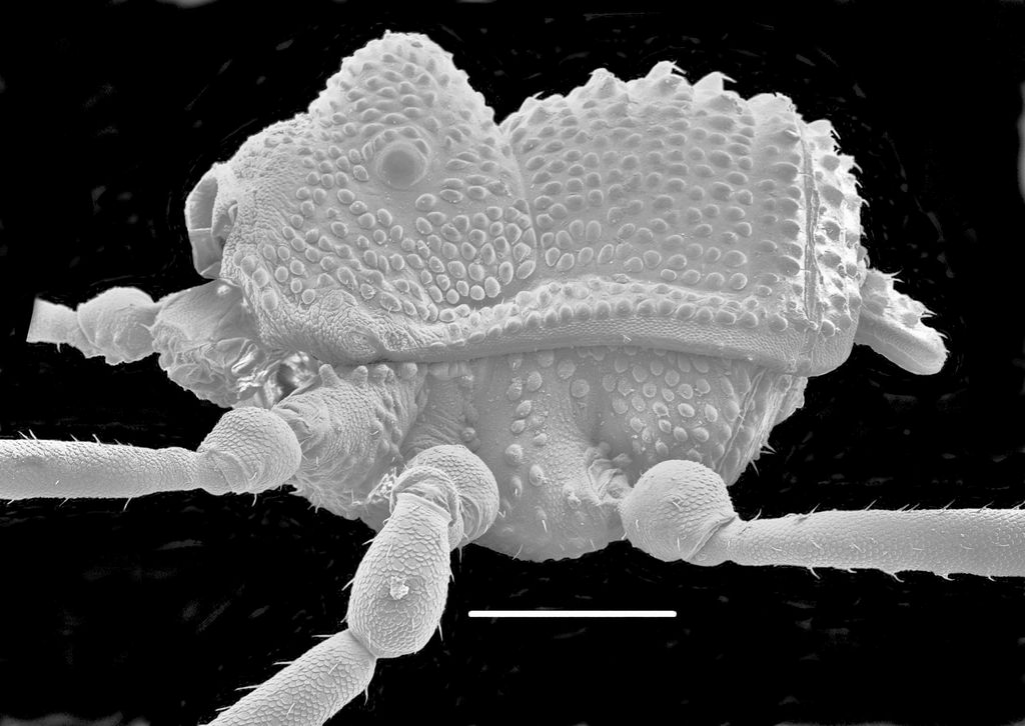
Biantidae, *Hovanoceros* sp., from Madagascar (FMNH AK 093), habitus (without chelicerae and pedipalps), lateral view. Photo by A.B. Kury (ABK). Scale bar = 0.5 mm.

**Figure 2.**
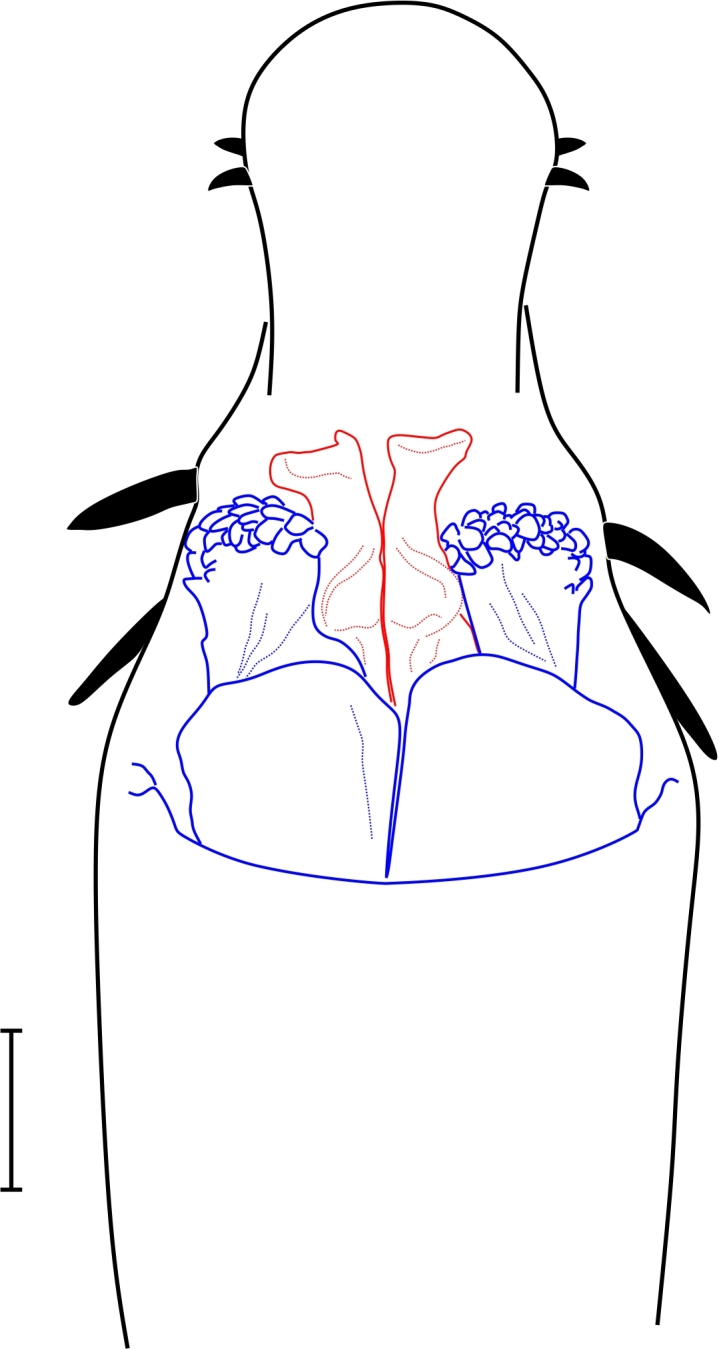
Biantidae, *Hovanoceros* sp. from Madagascar (FMNH AK 093) penis, distal portion, dorsal view. Drawing by ABK. Blue = titillators; red = conductors. Scale bar = 0.02 mm.

**Rationale for the new familial allocation.** External features include a huge protuberant ocularium and an unusual scutal armature, but they do not point conclusively to any specific family (See Fig. 1. Male genitalia presents an eversible stylus, flanked by a pair of rigid laminar conductors. The capsula externa consists of a bifid pair of soft titillators, with multiple digitiform apical lobules. This pattern doesn't present any known condition in Samoidae and perfectly matches that of Biantidae (Fig. 2, and is herein transferred into the family Biantidae.

***Malgaceros* Lawrence, 1959, new familial allocation**

*Malgaceros* Lawrence 1959: 79 [type species: *Malgaceros boviceps* Lawrence, 1959, by original designation].

***Malgaceros boviceps* Lawrence, 1959**

*Malgaceros boviceps* Lawrence 1959: 80.

**Type data.** 3 **♂** 1 **♀** syntypes (MNHN, not examined), MADAGASCAR, Nosy Be.

**Background and allocation.** *Malgaceros* shares the same history in the literature as *Hovanoceros*.

**Rationale for the new familial allocation.** It is clearly closely related *to Hovanoceros* as mentioned in the original description, and is here transferred into the same family Biantidae.

***Tetebius* Roewer, 1949, new familial allocation**

*Tetebius* Roewer 1949a: 288 [type species: *Tetebius latibunus* Roewer, 1949, by original designation].

**Historical systematic background.** *Tetebius* is a monotypic genus with a species from Mozambique, which was originally placed in Phalangodidae: Phalangodinae. It was removed from the Phalangodidae but not placed anywhere else by Staręga (1989). Then it was transferred to Samoidae by Staręga (1992).

**Figure 3.**
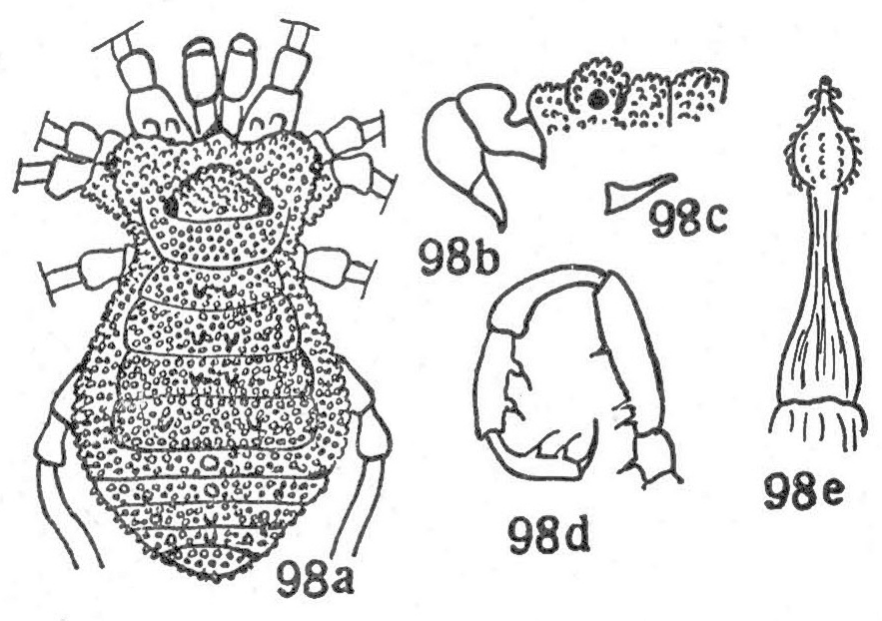
Biantidae, *Tetebius latibunus* Roewer, 1949, from Mozambique. From original description.

**Rationale for the new familial allocation.** *Tetebius latibunus* has not been examined by us. External features are inconclusive, but the sketch illustration of the penis as drawn by Roewer (1942a) in ventral view appears close to that of *Hovanoceros* (See Fig. 3), and so *Tetebius* is here transferred into the same family Biantidae.

### Cryptogeobiidae

As Cryptogeobiidae are members of the Gonyleptoidea, they are not treated in the Part 2 of this series, but rather in Part 5 (Lesser Gonyleptoidea).

***Tibangara* Mello-Leitão, 1940**

*Tibangara* Mello-Leitão 1940: 100 [type species: *Tibangara nephelina* Mello-Leitão, 1940, by original designation].

**Taxonomic background.** *Tibangara* was recently transferred from Phalangodidae to Cryptogeobiidae (Kury 2014).

***Tibangara soerenseni* (Soares & Soares, 1954) new comb., new familial allocation**

*Microminua soerenseni* Soares and Soares 1954: 503, fig. 13.

Gen. sp. W: Kury 2014: 11.

**Type data.** ♀ holotype (**MZSP** 842, examined), ♀ paratypes (**MZSP** 833, examined), from BRAZIL, Rio de Janeiro, Rio de Janeiro, Corcovado.

**Figure 4.**
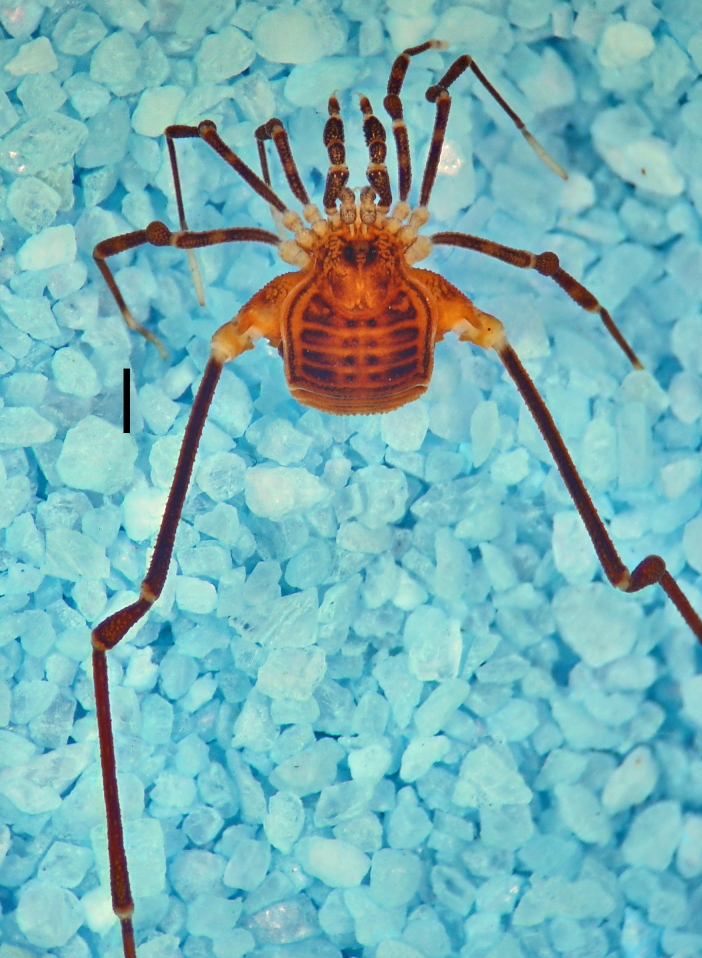
Cryptogeobiidae, *Tibangara soerenseni* (Soares & Soares, 1954) *comb. nov*. Non-type male (MNRJ 8184), from Rio de Janeiro, Brazil. Habitus, dorsal view. Photo by Daniele R. Souza (with permission).

**Rationale for the new familial and generic allocation of the species.** Males and females of this species have been examined from the type locality (as well as the types) and based both on external features as in male genitalia they are close relatives of *Tibangara nephelina*. Their similarity with Kimulidae is only superficial (Fig. 4).

### Cranaidae

**Cranaidae: Prostygninae**

***Llaguenia* Gen. nov.** /**new genus​**

**Type species.** *Zamora peruviana* Roewer, 1956.

**Etymology.** Generic name derives from Hacienda Llaguén in Peru, the collection locality of the type species.

**Diagnosis.** In both sexes, dorsal scutum outline alpha with coda notably elongate and slightly divergent. Mesotergum divided into four areas. Area I divided into left and right halves. Scutum and free tergites smooth and unarmed. Ocularium extremely narrow. Scutal groove U-shaped, notably elongate. Cheliceral hand of male swollen. Pedipalpal femur cylindrical, unarmed ventrally. Coxa IV outline widely surpasses dorsal scutum in dorsal view, and parallel to main body axis. Femur IV of male incrassate and armed with row of eight proventral spines. Penis ventral plate subrectangular, with lateral and apical wide concavities, three pairs of small macrosetae (MS) C, one pair of small MS D, and two pairs of very long MS A. Stylus sinuous, with slightly squared head, and well developed thumb-like dorsal process. *Cutervolus* and *Prostygnus* have a sexually dimorphic ocularium sexually dimorphic, hugely developed in males, scutal area III armed with powerful erect acuminate spines, free tergite III with paired spines, cheliceral hand immensely swollen, pedipalpal femur with ventral row of spines (*Prostygnus* only), MS A short, distal border of ventral plate straight (*Prostygnus*) or strongly convex (*Cutervolus*).

***Llaguenia peruviana* (Roewer, 1956), new comb.**

*Zamora peruviana* Roewer 1956: 436, figs. 8–9.

**Type data.** ♂ holotype (SMF RII 9701, examined), 1 ♀ paratype (SMF RII 9702, examined), PERU, La Libertad, Hacienda Llaguén, forest of Rejo Cargaruay, 2650 m.

**Figure 5.**
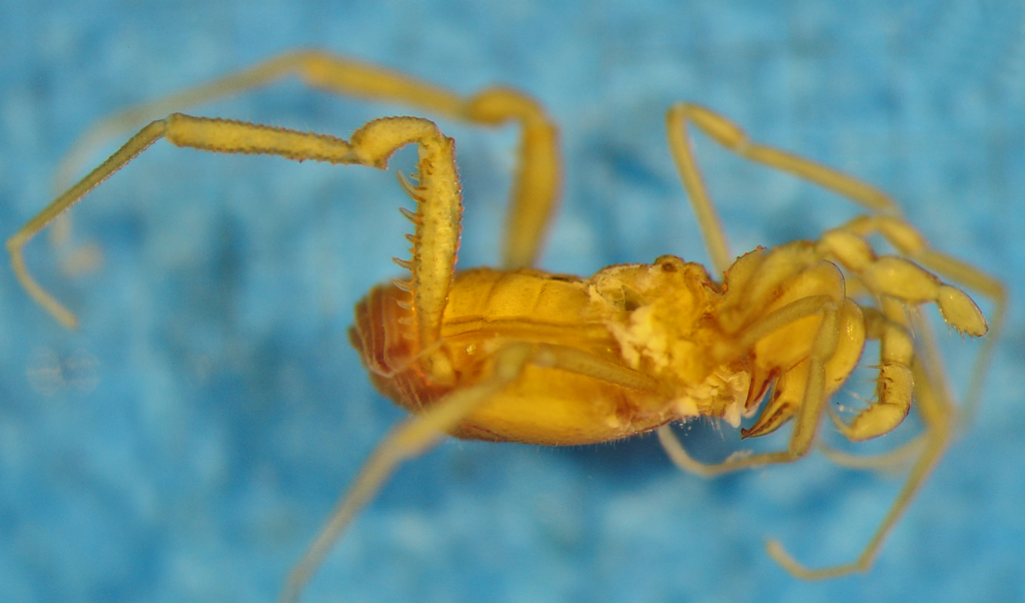
Cranaidae: Prostygninae, *Llaguenia peruviana* (Roewer, 1956), male holotype (SMF RII 9701) from Peru. Habitus, lateral view. Photo by A. Pérez-González (APG).

**Figure 6.**
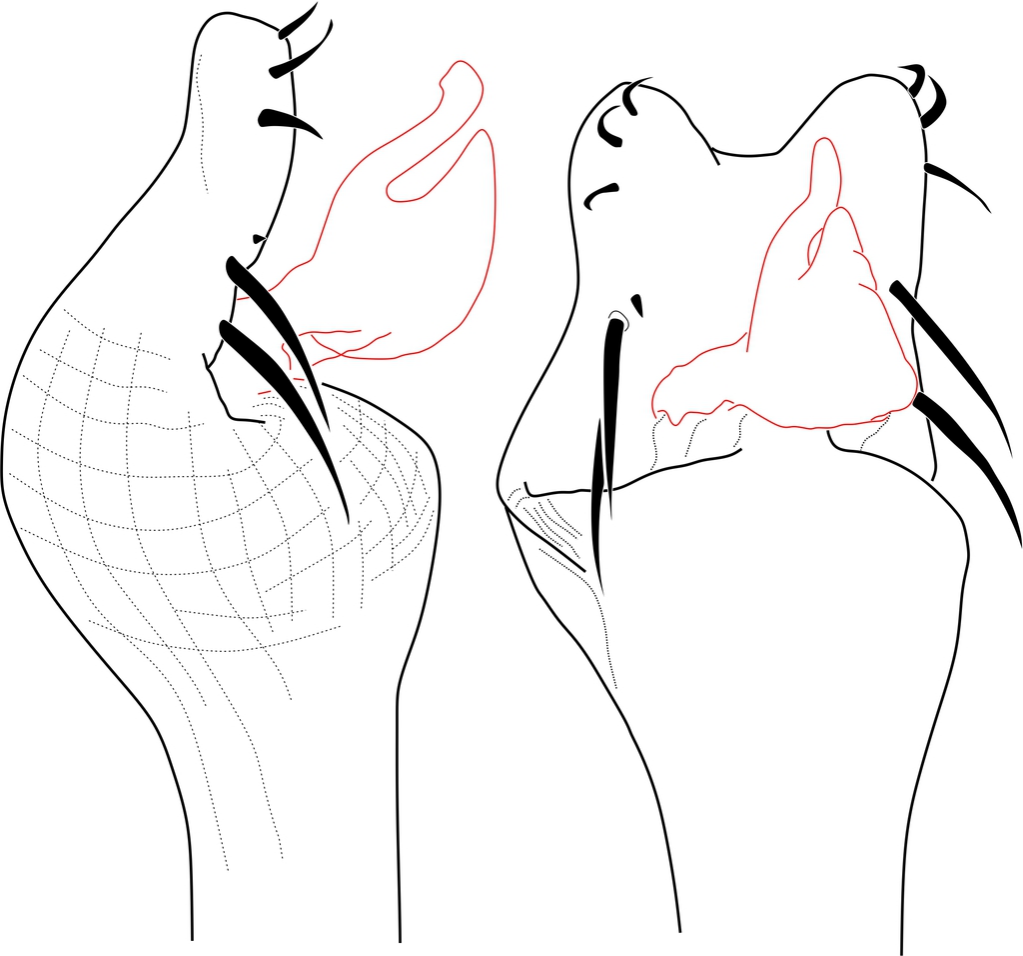
Cranaidae Prostygninae, *Llaguenia peruviana* Roewer, 1956, male holotype (SMF RII 9701), from Peru. Pars distalis of penis, lateral and dorso-lateral views. Drawing by APG/ABK. Red = glans.

**Rationale for the new familial allocation.** The subfamily Prostygninae is currently in Cranaidae (Kury 2012). The holotype of *Zamora peruviana* has been examined, and although it has an apparent metasarcid facies (Fig. 5), male genitalia strongly resembles the ones depicted for Prostygninae in Kury (2012) (Compare Fig. 6). *Zamora* is no longer considered to be a cranaid (see Kury and Villarreal M. 2015), and *L. peruviana* does not have any special similarity to grant its permanence in that genus. Consequently, this species does not belong in any of the superfamilies so far treated in this planned series of papers, nor should remain *incertae sedis* anymore, and hereby formally transferred to Gonyleptoidea: Cranaidae (Prostygninae).

### Assorted Grassatores

Phalangodidae was once a great repository of diverse species (*e.g.* Roewer 1912), with no clear monophyly. Revision is increasingly showing that many genera originally described in Phalangodidae: Phalangodinae the mid-20th century should be allocated elsewhere (*e.g.* Staręga 1989), diminishing this family to a natural Holarctic natural core. Of these "false phalangodines", *Beloniscus* and closely allied genera are currently under study for inclusion into another family, and so are not treated here.

**Grassatores*incertae sedis***

***Bebedoura* Roewer, 1949**

*Bebedoura* Roewer 1949c: 56 [type species: *Bebedoura rugosa* Roewer, 1949, by original designation].

***Bebedoura rugosa* Roewer, 1949**

*Bebedoura rugosa* Roewer 1949c: 56, figs 108a-d; Kury 2003: 201.

**Type data.** ♂ holotype (SMF RII 6897/17, examined), from BRAZIL, Pernambuco, Bebedouro (wrongly cited as in "São Paulo" state in).

**Historical systematic background.** *Bebedoura* was originally included in Phalangodidae: Tricommatinae. A recent cladistic analysis of this group (Kury 2014) detected that Tricommatinae as then recognised consisted of two divergent groups (Gonyleptidae: Tricommatinae versus Cryptogeobiidae) plus some extraneous genera of gonyleptids of uncertain placement, such as *Bebedoura*. However, Kury was inconsistent because he listed *Bebedoura* among the Gonyleptidae *incertae sedis*, and at the same time commented "probably Escadabiidae", which are mutually excluve placements.

**Figure 7.**
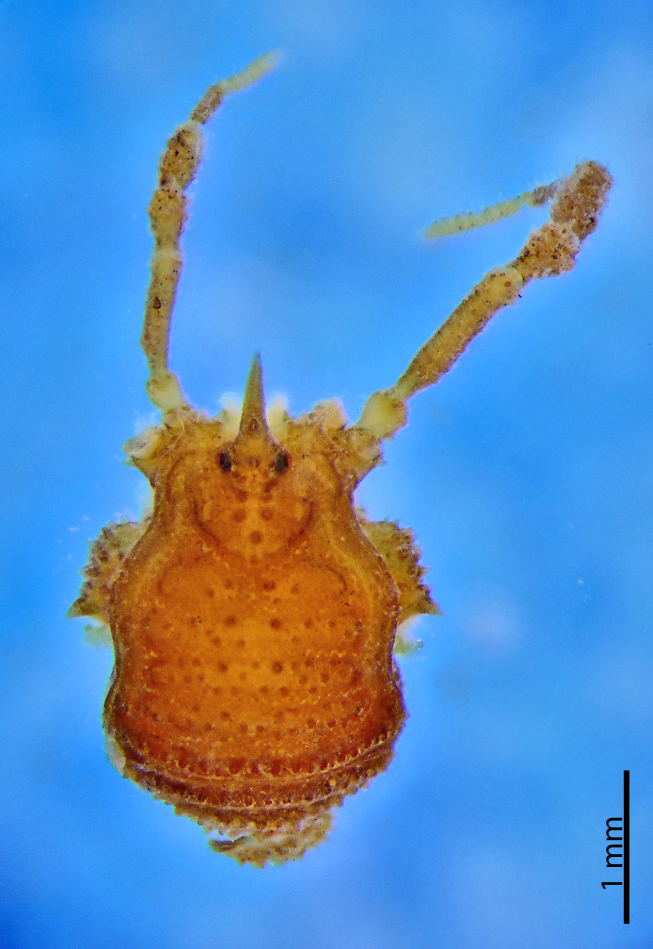
Grassatores, *Bebedoura rugosa* Roewer, 1949, female holotype (SMF RII 6897/17), from Pernambuco, Brazil. Habitus, dorsal view. Photo by Tiago N. Bernabé. Scale bar = 1 mm.

**Figure 8.**
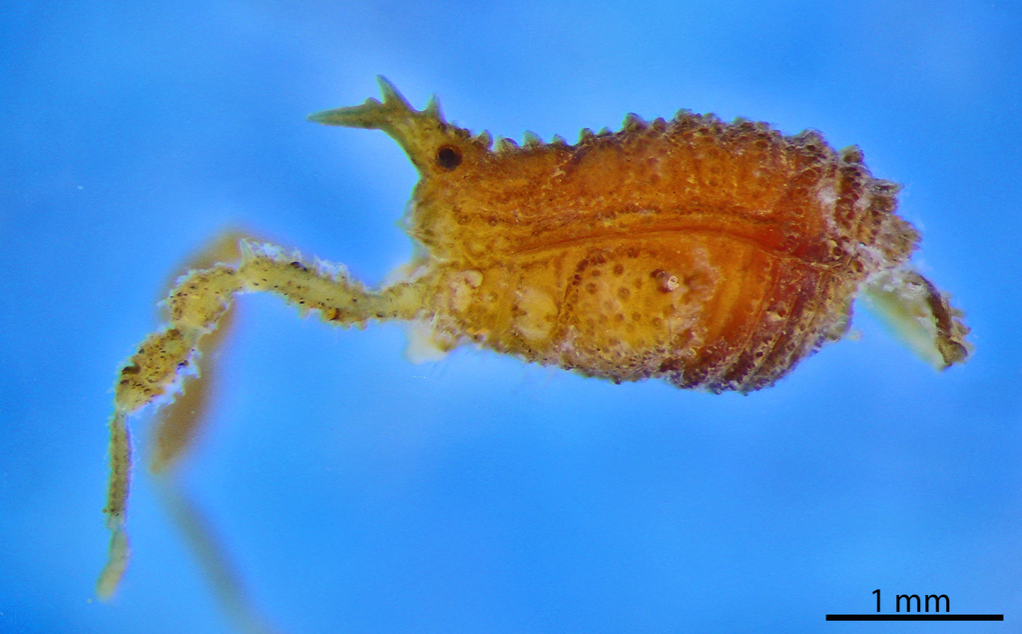
Grassatores, *Bebedoura rugosa* Roewer, 1949, female holotype (SMF RII 6897/17), from Pernambuco, Brazil. Habitus, lateral view. Photo by Tiago N. Bernabé (with permission). Scale bar = 1 mm.

**Rationale for the removal from Tricommatinae.** The female holotype of *Bebedoura rugosa* has been studied by ABK (see Figs 7, 8), but it failed to provide any evidence for a sure familial placement. The ocularium is seamlessly prolonged into a huge forward bent cone with a secondary branch found only in Podoctidae. Leg IV vaguely resembles some Zalmoxidae because of its curved femur armed with a retrodistal apophysis (females usually do not possess those in many families). The Cryptogeobiidae which possess similar ocularia (*e.g., Pseudopachylus*, *Tibangara*) do not share any other special similarity with *Bebedoura*. There are some Gonyleptidae (Pachylinae) with females also known to bearmed on leg IV, while a divided area I occurs in many Gonyleptoidea. Consequently, *Bebedoura* is here formally transfered to Grassatores *incertae sedis* (See Figs 7, 8).

***Microconomma* Roewer, 1915**

*Microconomma* Roewer 1915: 12; Roewer 1949c: 58 [type species: *Microconomma armatipes* Roewer, 1915, by monotypy].

***Microconomma armatipes* Roewer, 1915**

*Microconomma armatipes* Roewer 1915: 12; Roewer 1927: 84, fig. 81; Roewer 1949c: 58.

**Type data.** ♂ holotype (SMF RI 1131, not examined), from CAMEROON, Kamerun Mountains, Bakossu, 400 m.

**Historical systematic background.** Monotypic genus, with one species from Cameroon. *Microconomma* was originally placed in Phalangodinae. It was transferred by Roewer (1949c) to the subfamily Samoinae (since revised as Samoidae). Staręga (1989) listed it in an as then undescribed family, which only much later would be described as Pyramidopidae (Sharma et al. 2011b). But Sharma et al. (2011a) kept this genus in Samoidae.

**Rationale for the removal from Samoidae.** At the moment we have no evidence to support either previously suggested assignment and so transfer this genus to Grassatores *incertae sedis*. The original description is too superficial to allow any judgement, but the species in question, as illustrated, doesn't fit the typical Samoidae habitus as defined by Pérez-González and Kury (2007b). On the other hand, the well-defined cheliceral bulla points away from the Samoidae, and more to the Zalmoxoidea. Therefore, it is more sensible for now to list this taxon as Grassatores incertae sedis.

***Stygnomimus* Roewer, 1927**

*Stygnomimus* Roewer 1927: 305 [type species: *Stignomimus conopygus* Roewer, 1927 by monotypy].

***Stygnomimus conopygus* Roewer, 1927**

*Stignomimus conopygus* Roewer 1927a: 305.

**Type data.** ♂ holotype (SMF RII/63/1, examined), [Indonesia, Riau Islands], Riouw Archipelago.

***Stygnomimus malayensis* Suzuki, 1969**

*Stignomimus malayensis* Suzuki 1969: 32.

**Type data. ♀** holotype (not examinined)Templer Park, Malaysia.

**Historical systematic background** The original assignment of this Indomalayan genus to Stygnommatidae by Roewer (1927) was made based on apparently trivial characters such as the absence of a common ocularium. Suzuki (1969) provided a beautiful description of a second species, unfortunately known only from a female.

**Rationale for the removal from Stygnommatidae.** There is no positive evidence yet to assign the genus to Stygnommatidae or even to Samooidea. Pérez-González (2006) suggested it should be set apart until further study, and this opinion is here formalized, so transferred to Grassatores *insertae sedis*.

### Samoidae

***Bichito* González-Sponga, 1998, new familial assignment**

*Bichito* González-Sponga 1998: 29 [type species: *Bichito pijiguaoensis* González-Sponga, 1998, by original designation].

***Bichito pijiguaoensis* González-Sponga, 1998**

*Bichito pijiguaoensis* González-Sponga 1998: 29, figs 8–14; Kury 2003a: 23.

**Type data.** ♂ holotype (MAGS 1222a, not examined); 1 ♀ paratype (MAGS 1222b, not examined); 5 ♂ 5 ♀ 3 juv. paratypes (MAGS, not examined), from VENEZUELA, Bolívar, Cedeño: Near bauxite mines of Los Pijiguaos, 06°35’20’’N, 66°45’12’’W, 80 m.

**Historical systematic background.** Originally described in Phalangodinae, removed to Grassatores *incertae sedis* by Kury (2003).

**Figure 9.**
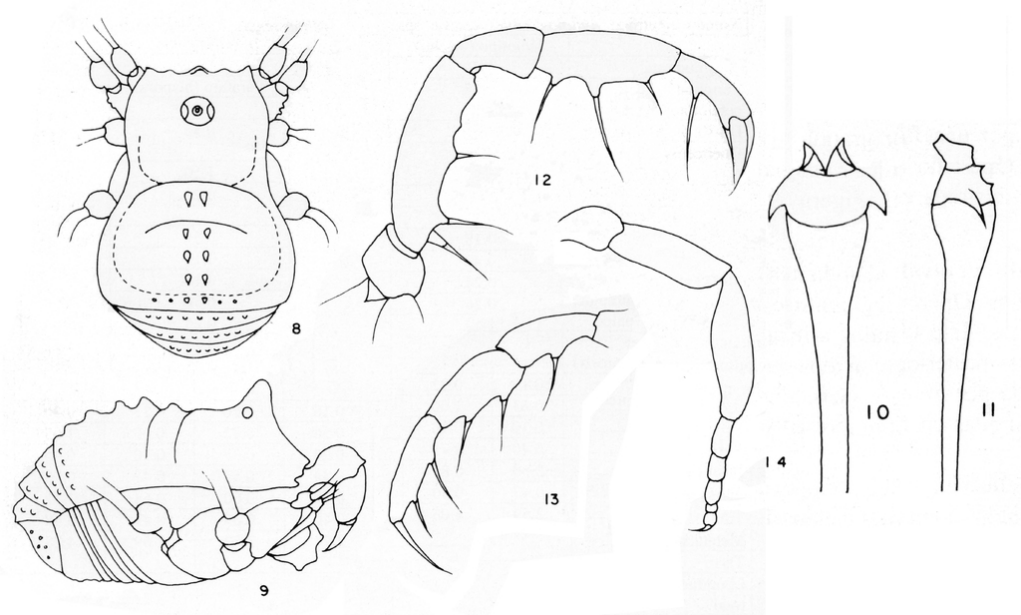
Samoidae, *Bichito pijiguaoensis* González-Sponga, 1998, from Venezuela. From original description.

**Rationale for the new placement.** The original drawings of the male genitalia are poorly detailed, but they allow recognition of an everted penis with two conductors, capsula interna and glans without stragulum or modified follis (Fig. 9). This genital morphology, combined with the remarkable sexual dimorphism of enlarged metatarsus III in males supports the transferrence of this species to Samoidae (Fig. 9).

***Microminua* Sørensen, 1932, new familial assignment**

*Microminua* Sørensen in Henriksen 1932: 245 [type species: *Microminua parvula* Sørensen, 1932, by monotypy].

*Cornigera* Gonzalez-Sponga 1987: 86 [type species: *Cornigera flava* González-Sponga, 1987, by original designation]. **NEW SYNONYMY**

**Rationale for the new placement.** The new family placement is fully supported by the male genital groundplan where the truncus is cylindrical, without a well-defined ventral plate as in Gonyleptoidea, with pars distalis, compressed dorsoventrally, not differentiated from pars basalis by any remarkable groove or constriction and laterally armed with strong spatulate (foliar) spines. The capsula interna is eversible and formed by a pair of conductors completely fused. The follis is not modified into a stragulum and not observed externally. This genital morphology matches the penial groundplan described for Samoidae by Pérez-González and Kury (2007b). The absence of the Kimulidae penial morphology where pars distalis has a very peculiar form with the lamina ventralis surrounding the capsula interna (conductors + stylus) avoids keeping this genus in Kimulidae.

***Microminua flava* (González-Sponga, 1987), new comb.**

*Cornigera flava* Gonzalez-Sponga 1987: 86, figs. 58-63.

**Type data.** ♂ holotype (MCNC, not examined), VENEZUELA, Miranda, Zamora: Salmerón, 250 m.

***Microminua parvula* Sørensen, 1932**

*Microminua parvula* Sørensen in Henriksen 1932: 245, fig. 8.

**Type data.** 30 ♂ ♀ syntypes (ZMUC; subsample at SMF RII/6237/165-4, examined), from VENEZUELA, Distrito Federal, Hacienda La Moka, right margin of Siquire River, 10 km NE Santa Lucia on road Caracas-Santa Lucia.

**Historical systematic background.** *Microminua* was originally established in Minuidae by Sørensen (Henriksen 1932) along with the type species *Microminua parvula* Sørensen, 1932 from Venezuela. It was removed to Phalangodidae: Phalangodinae by Mello-Leitão (1938). Soares and Soares (1954) described a second species *Microminua soerenseni* Soares & Soares, 1954 from Rio de Janeiro, Brazil, based on a single female. Gonzalez-Sponga (1987) described in Phalangodinae the monotypic genus *Cornigera*, along with the species *Cornigera flava*, from Miranda, Venezuela. Kury (2003) transferred *Cornigera* from Phalangodinae to Samoidae, and *Microminua* from Phalangodinae back to Minuidae.

**Figure 10.**
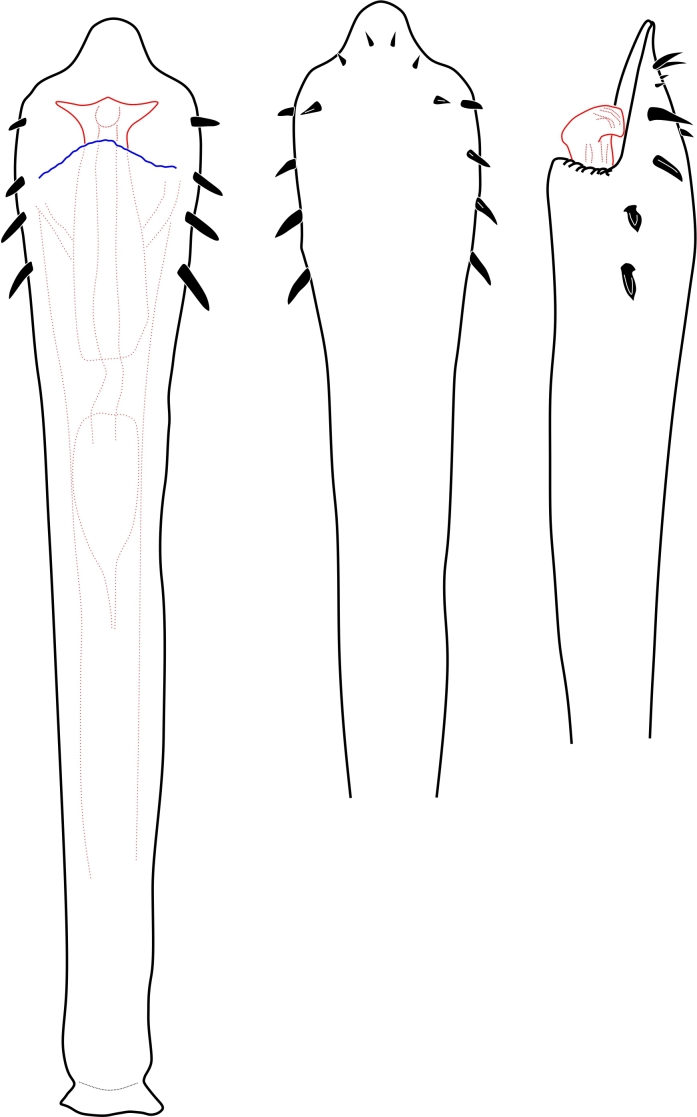
Samoidae, *Microminua parvula* Sørensen, 1932, genitalia of male syntype (SMF RII 6237), from Venezuela. Drawing by APG/ABK. Blue = capsula externa, red = capsula interna.

**Figure 11.**
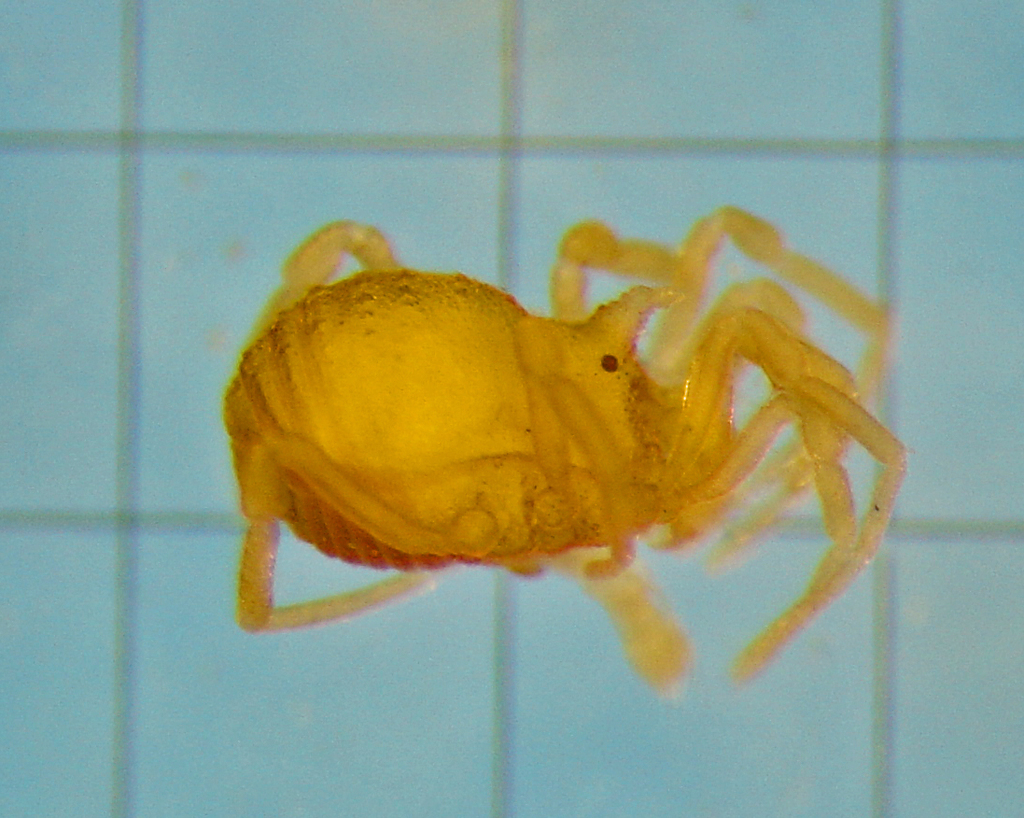
Samoidae, *Microminua parvula* Sørensen, 1932, male syntype (SMF RII 6237), from Venezuela. Photo by APG.

**Figure 12.**
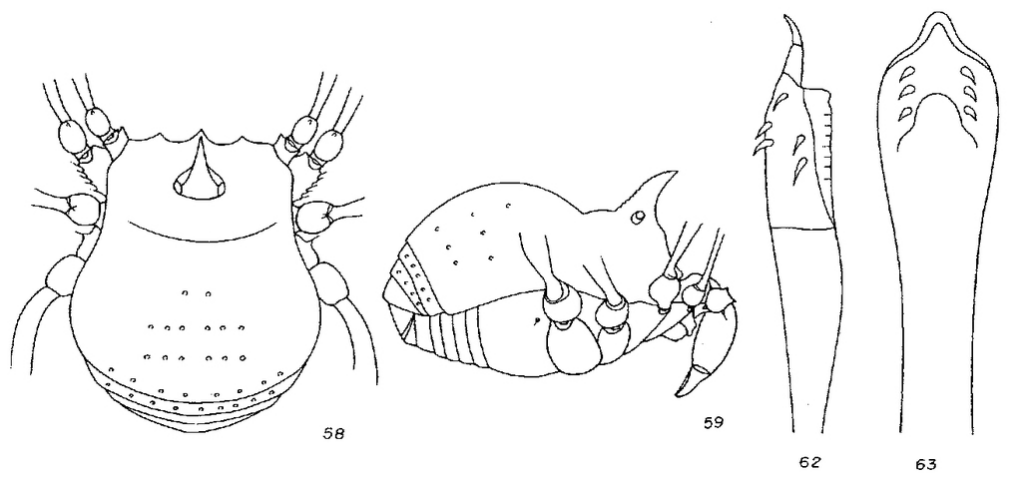
Samoidae, *Cornigera flava* González-Sponga 1987, from original description.

**Rationale for the generic synonymy.** The male genitalia of the type material of *Microminua parvula* (syntypes in **SMF** and **ZMUC** (See Fig. 10) have been examined for the first time, and they match closely to those of *Cornigera flava* (Fig. 12. Also the external features (Fig. 11 of the type species of both alleged genera are a near perfect match, not to mention their geographic distribution, as both species are from Venezuelan coastal range.Consequently, *Cornigera* is considered a junior synonym of *Microminua*, with two valid species in the family Samoidae.

***Niquitaia* González-Sponga, 1999, new familial assignment**

*Niquitaia* González-Sponga 1999: 64 [type species: *Niquitaia convexa* González-Sponga, 1999, by original designation].

***Niquitaia convexa* González-Sponga, 1999**

*Niquitaia convexa* González-Sponga 1999: 64, figs 19–24; Kury 2003: 246.

**Type data.** ♂ holotype (MAGS 1254a, not examined); 1 ♀ paratype (MAGS 1254b, not examined); 1 ♂ 3 ♀ paratypes (MAGS, not examined), VENEZUELA, Trujillo, Boconó: km 4 road Boconó-Niquitao, 1400 m.

**Historical systematic background.** *Niquitaia* was originally established in Phalangodidae: Phalangodinae along with the type species *Niquitaia convexa* from Venezuela. It was removed to Zalmoxidae by Kury (2003).

**Figure 13.**
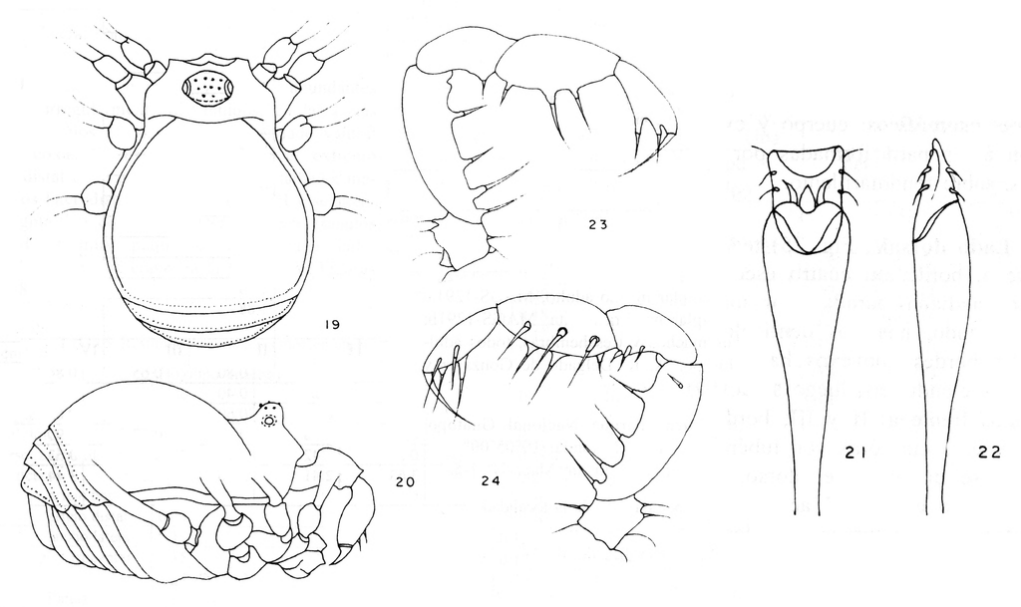
Samoidae, *Niquitaia convexa* González-Sponga, 1999, from Venezuela. From original description.

**Rationale for the new placement.** The external morphology is very similar to *Kalominua* Sørensen, 1932 (Samoidae): body as an asymmetrical hourglass, anterior half of scutum much shorter, posterior half rounded, laterally convex appearance, male genitalia without stragulum, with pars distalis flattened dorso-ventrally with ventral region wide, undivided and armed with strong macrosetae (Fig. 13). All of the above characters justify the inclusion of *Niquitaia* in Samoidae.

### Zalmoxidae

The Zalmoxidae are here augmented by the inclusion of two genera originally described in Phalangodidae and one species, originally included in a genus of Samoidae.

***Heteroscotolemon* Roewer, 1912 new familial assignment**

*Heteroscotolemon* Roewer 1912: 150; Kury 2003: 24 [type species: *Heteroscotolemon australis* Roewer, 1912, by monotypy].

***Heteroscotolemon australis* Roewer, 1912**

*Heteroscotolemon australis* Roewer 1912: 151, fig. 34; Kury 2003: 24.

**Type data.** ♀ [originally reported as ♂] holotype (SMF RI/207, examined), from “Guayana: Nieder-Oyopock” [FRENCH GUYANA, Lower Oyapock River, which marks the fro ntier with Brazil].

**Historical systematic background.** *Heteroscotolemon* was originally included in Phalangodinae, then transferred to Grassatores *incertae sedis* by Kury (2003).

**Figure 14.**
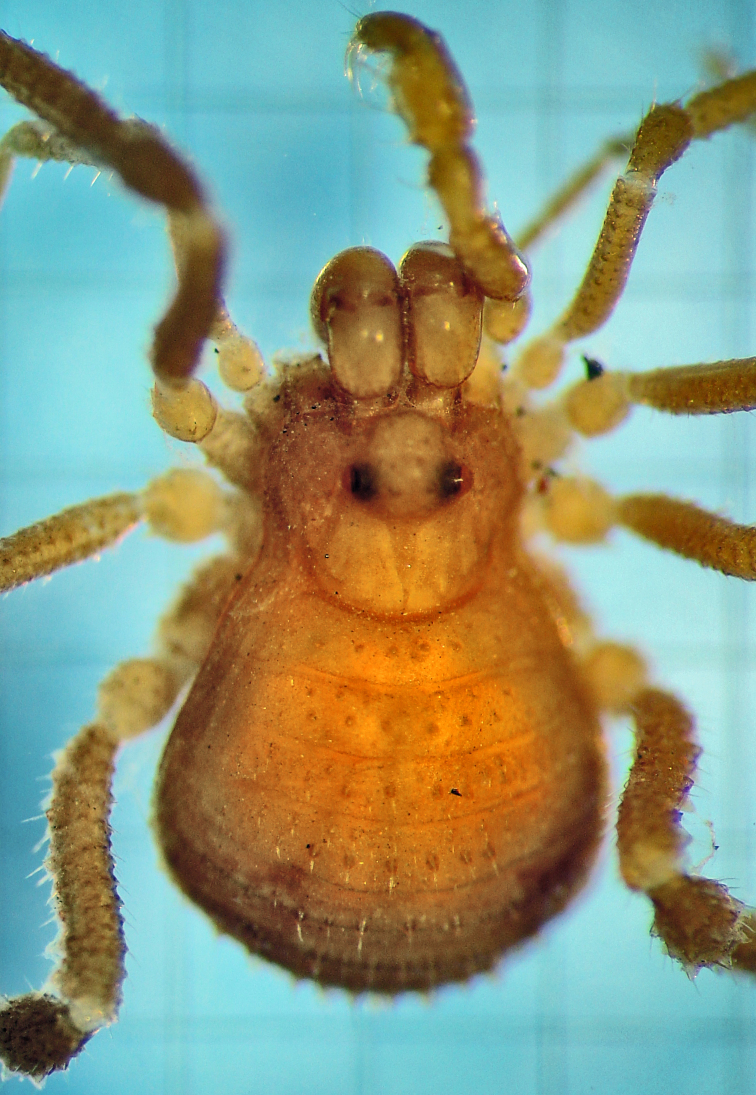
Zalmoxidae, *Heteroscotolemon australis* Roewer, 1912, female holotype (SMF), habitus, dorsal view. Photo by APG.

**Figure 15.**
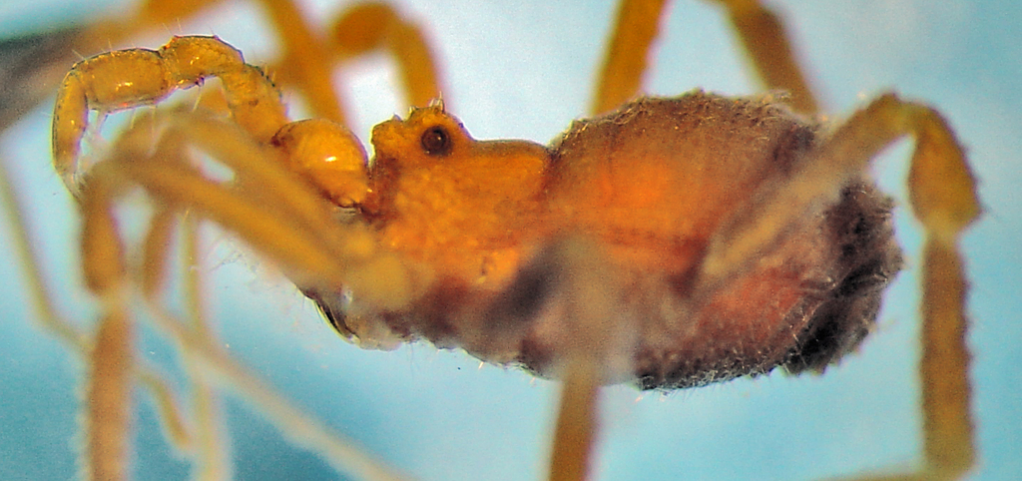
Zalmoxidae, *Heteroscotolemon australis* Roewer, 1912, female holotype (SMF), habitus, lateral view. Photo by APG.

**Rationale for the new placement.** Evidence for the new placement as a large zalmoxid comes from the backward pointed scutal grooves, bimerous distitarsus I, strongly armed pedipalpal trochanter and femur. All other Laniatores recorded from French Guyana are either Gonyleptoidea, which typically have distitarsus I trimerous, or Stenostygninae, which do not have a common ocularium. *Heteroscotolemon australis* is very different from *Parascotolemon ornatum* Roewer, 1912, a typical local zalmoxid (See Figs 14, 15). However, here *Heteroscotolemon* is formally transferred to Zalmoxidae, pending its further study.

***Minuides* Sørensen, 1932**

*Minuides* Sørensen in Henriksen 1932: 237 [type species: *Minuides setosa* Sørensen, 1932, by monotypy].

***Minuides americanus* (Roewer 1952), new comb.**

*Zalmoxista americana* Roewer 1952: 40.

**Type data.** ♀ holotype (SMF RII 10226/240, not examined), PERU, Pasco, Laguna Punrun, 4400 m, near Cerro do Pasco in drainage of Junin Lake.

**Historical systematic background.** Roewer (1949a) originally created *Zalmoxista* in Phalangodinae to include two species from Australia and one from New Caledonia. Later he added a fourth species from Peru (*Zalmoxista americana* Roewer, 1952), in a very brief unillustrated description. Kury (2003) treated this Peruvian “*Zalmoxista*” *americana* as Grassatores *incertae sedis*. The type species, *Phalangodes australis* Sørensen, 1886, was transferrred to Samoidae by Pérez-González and Kury 2007a, but the other two Australasian species were included in *Zalmoxis* by Sharma (2012).

**Rationale for the new placement.** The identity of "*Zalmoxista" americana* is highly doubtful, but it may be recognized as a Zalmoxidae. Another zalmoxid genus which has the same tarsal counts and the same conformation of scutal areas is *Minuides*, which currently includes several Neotropical species of doubtful monophyly. Therefore this species is here included in *Minuides* (Zalmoxidae) pending further study.

***Neobabrius* Roewer, 1949, new familial assignment**

*Neobabrius* Roewer 1949c: 19 [type species: *Neobabrius parvulus* Roewer, 1949, by original designation].

***Neobabrius parvulus* Roewer, 1949**

*Neobabrius parvulus* Roewer 1949c: 19, figs 17a-c.

**Type data.** ♂ holotype, 1 ♂ 1 ♀ paratypes (SMF RII 3138/79, not examined), from INDONESIA, Jawa Timur, Bawean Island.

**Historical systematic background.** This monotypic genus was originally in Phalangodinae and represents a neglected taxon. Goodnight and Goodnight (1957) synonymized a great number of genera with *Zalmoxis*, but overlooked *Neobabrius*. Staręga (1989) when resurrecting Zalmoxidae, gave a list of Phalangodinae of obscure systematic position, but *Neobabrius* was again not included. It was neither mentioned in Sharma et al. (2011a).

**Figure 16.**
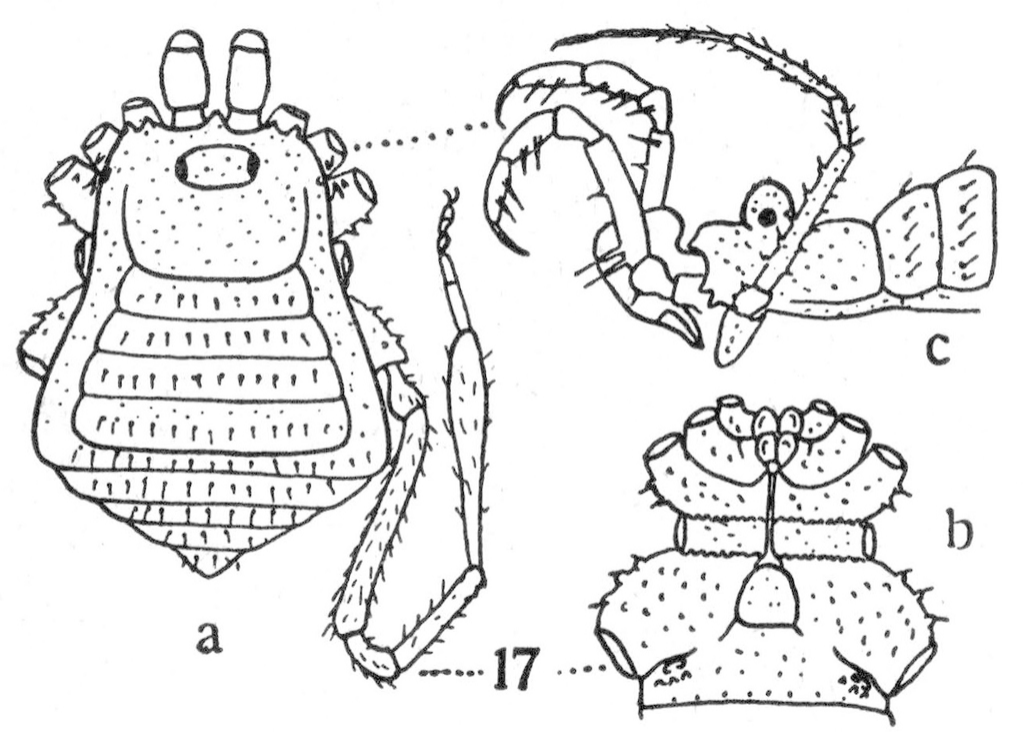
Zalmoxidae, *Neobabrius parvulus* Roewer, 1949, from Indonesia, Bawean Island. From original description.

**Figure 17.**
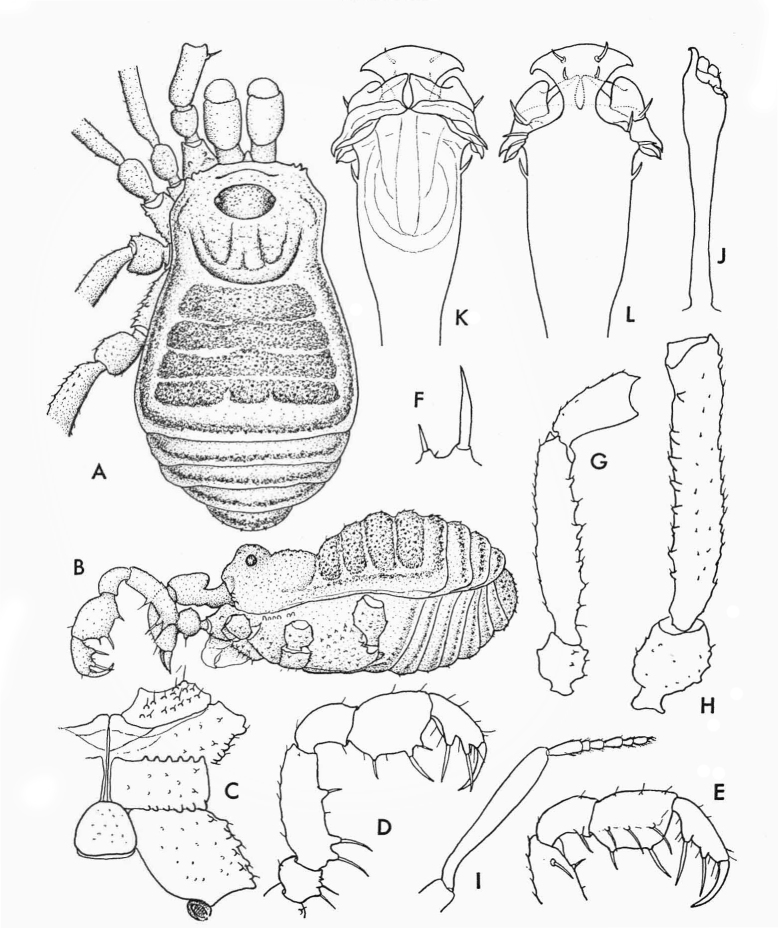
Zalmoxidae, *Zalmoxis heynemani* Suzuki, 1977, from Philippines, Mindanao. From original description.

**Rationale for the new placement.** Roewer's description of *Neobabrius parvulus* (Fig. 16) allows us to recognize a body structure extremely similar to *Zalmoxis heynemani* Suzuki, 1977 (Fig. 17), and as a typical zalmoxid due the genitalic features, Fig. 17 outline of dorsal scutum, shapes of areas and ocularium, proportion of coxae and stigmatic areas, spination of pedipalps, and metatarsus IV incrassate. Therefore *Neobabrius* is here transferred to Zalmoxidae. Old World Zalmoxidae are currently included in only one genus *Zalmoxis*, but we do not wish to propose any additional changes yet, leaving the Indonesian *Neobabrius* as a separate valid genus of Zalmoxidae for now.

***Soledadiella* González -Sponga, 1987**

*Soledadiella* Gonzalez-Sponga 1987: 304 [type species: *Soledadiella barinensis* González-Sponga, 1987, by original designation].

***Soledadiella roraima* (Goodnight and Goodnight, 1943), new comb.**

*Crosbyella roraima* Goodnight and Goodnight 1943: 1, figs. 1–4.

*Pellobunus roraima*:Goodnight and Goodnight 1947​: 5.

*“Crosbyella” roraima*: Kury 2003: 241.

**Type data.** ♀ holotype (AMNH, examined only by photograph), from [VENEZUELA, Bolívar], “Rondon Camp, Mt. Roraima, 6900 feet.”

**Historical systematic background.** This species from the border of Brazil and Venezuela has originally been assigned to *Crosbyella* (Phalangodinae) and then *Pellobunus* (Samoidae). Kury (2003) recognized it as a zalmoxid, but refrained from indicating a particular genus. There are a great number of described Venezuelan species of Zalmoxidae, mostly placed in monotypic genera of no meaning.

**Figure 18.**
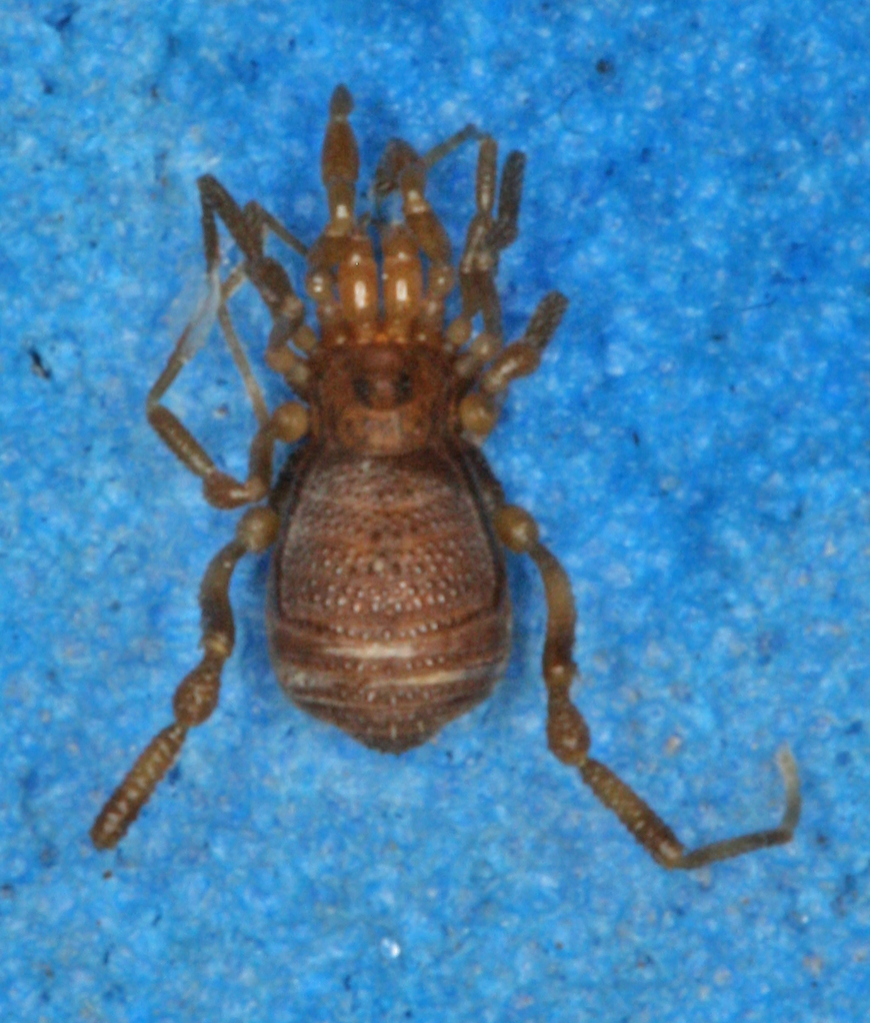
Zalmoxidae, *Soledadiella roraima* (Goodnight & Goodnight, 1943), female holotype (AMNH), habitus, dorsal view. Photo by Ricardo Pinto-da-Rocha (with permission).

**Figure 19.**
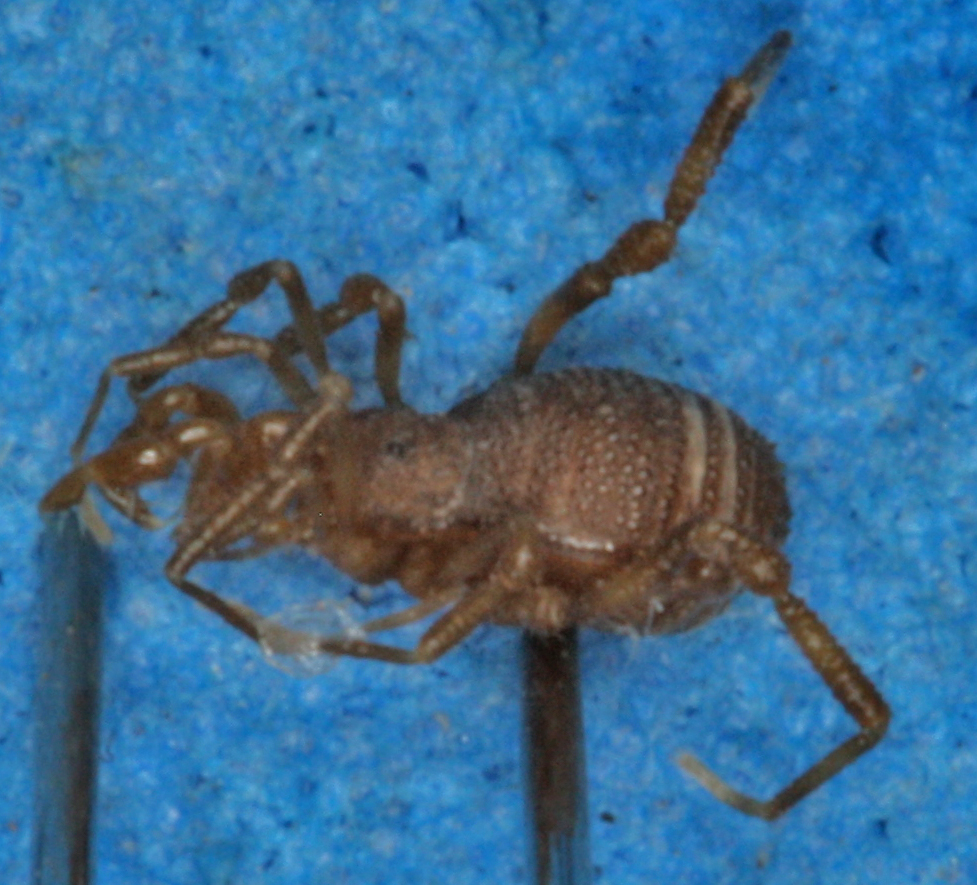
Zalmoxidae, *Soledadiella roraima* (Goodnight & Goodnight, 1943), female holotype (AMNH), habitus, dorso-lateral view. Photo by Ricardo Pinto-da-Rocha (with permission).

**Rationale for the placement.** Examining two pictures of the female holotype of *C. roraima* (in AMNH, photos courtesy of R. Pinto-da-Rocha), suggests placement in the genus *Soledadiella*: Ocularium not greatly developed and situated far from margin of carapace; abdominal scutum much larger than carapace, widening posteriorly, with convex sides without constriction, its posterior border straight; scutal area I slightly longer than each of the others; scutal grooves gently curved, pointing backwards; scutal areas and free tergites densely covered by coarse rounded granules, while carapace is smooth. (See Figs 18, 19). The tarsal counts of *Soledadiella roraima* nov. comb. are 4/6/5/6, while the formula for typical *Soledadiella* is 5/6/5/6. However, here *S. roraima* is treated as *Soledadiella* (Zalmoxidae).

***Zalmoxis* Sørensen, 1886**

*Zalmoxis* Sørensen 1886: 64 [type species: *Zalmoxis robusta* Sørensen, 1886 by subsequent designation of Roewer (1949c)].

*Zalmoxissus* Roewer 1949b: 143 [type species: *Zalmoxis tristis* Thorell, 1891, by original designation]. **NEW SYNONYMY**

**Historical systematic background.**​ Roewer (Roewer 1949c, Roewer 1949a) founded innumerous Palaeotropic genera close to *Zalmoxis* based on minimal variations of scutal armature and tarsal segmentation.

**Fundamentation of the synonymy.** All of those but the monotypic *Zalmoxissus* were synonymized under *Zalmoxis* by Sharma et al. (2011a), or Sharma (2012). We believe it was an oversight (especially because the name was published in a short, obscure paper), as *Zalmoxissus tristis* (Thorell, 1891) matches the current diagnosis of *Zalmoxis* and therefore we also propose *Zalmoxissus* as a further junior synonym of *Zalmoxis* (Zalmoxidae).

***Zalmoxis sorenseni* Simon, 1892 original spelling restored**

*Zalmoxis sorenseni* Simon 1892: 44, pl. 2, figs. 7–8.

*Zalmoxis soerenseni* [unjustified emended spelling]: Roewer 1912: 129; Sharma et al. 2011a: 53.

**Comment.** *Zalmoxis sorenseni* Simon, 1892 appears in the literature wrongly as *Zalmoxis soerenseni* Simon, 1892. The change of spelling proposed by Roewer 1912 to better reflect the Latin rendering of the Danish name Sørensen, is an incorrect subsequent spelling. ICZN (article 32.5.1.) says: "If there is in the original publication itself, without recourse to any external source of information, clear evidence of an inadvertent error, such as a *lapsus calami* or a copyist's or printer's error, it must be corrected. Incorrect transliteration or latinization, or use of an inappropriate connecting vowel, are not to be considered inadvertent errors."

***Zalmoxissus tristis* (Thorell, 1891)**

*Zalmoxis tristis* Thorell 1891: 750.

*Zalmoxida tristis*: Roewer 1912: 134.

**Type data.** ♂(?) holotype (MCSN) from PAPUA NEW GUINEA, National Capital District, Yule Island (“Yule-Roro”).

### Zalmoxoidea (no familial inclusion)

***Phalangodella* Roewer, 1912, new superfamilial assignment**

*Phalangodella* Roewer 1912: 160 [type species: *Phalangodella aequatorialis* Roewer, 1912, by monotypy].

*Erxlineia* Mello-Leitão 1942: 315 [original incorrect spelling; *lapsus calami*].

*Exlineia* Mello-Leitão 1943: 2 [type species: *Exlineia milagroi* Mello-Leitão, 1942, by original designation]. Syn. nov.

*Langodinus* Mello-Leitão 1949: 7 [type species: *Langodinus flavipes* Mello-Leitão, 1949, by original designation]. Syn. nov.

*Cochirapha* Roewer 1949c: 40 [type species: *Cochirapha rugipes* Roewer, 1949, by original designation]. Syn. nov.

*Phalpuna* Roewer 1949c: 41 [type species: *Phalpuna urarmata* Roewer, 1949, by original designation]. Syn. nov.

**Historical systematic background.** The monotypic genus *Phalangodella* was originally placed in Phalangodidae Tricommatinae. It was transferred to Grassatores *incertae sedis* by Kury (2003). A number of species have been subsequently described under *Phalangodella*, but they are all currently included in Cryptogeobiidae (Kury 2014). *Exlineia* Mello-Leitão, 1942 and *Cochirapha* Roewer, 1949c were originally placed in Phalangodidae: Phalangodinae. Both were transferred to Zalmoxidae by Kury (2003). *Langodinus* Mello-Leitão, 1949 and *Phalpuna* Roewer, 1949c were originally placed in Phalangodidae: Phalangodinae. Both were removed to Grassatores *incertae sedis* by Kury (2003).

**Figure 20.**
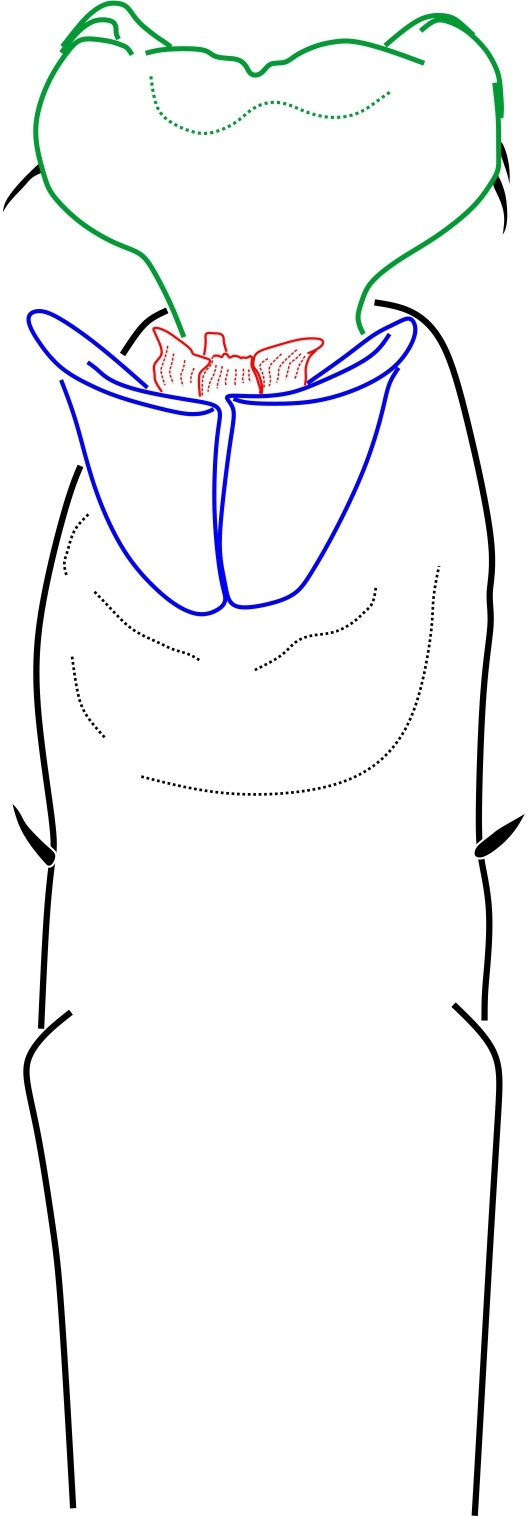
Zalmoxoidea, *Phalangodella* sp., male (MNRJ 2438) from Ecuador. Penis, expanded, dorsal view. Drawing by APG/ABK. Blue = stragulum; green = rutrum; red = capsula interna.

**Figure 21.**
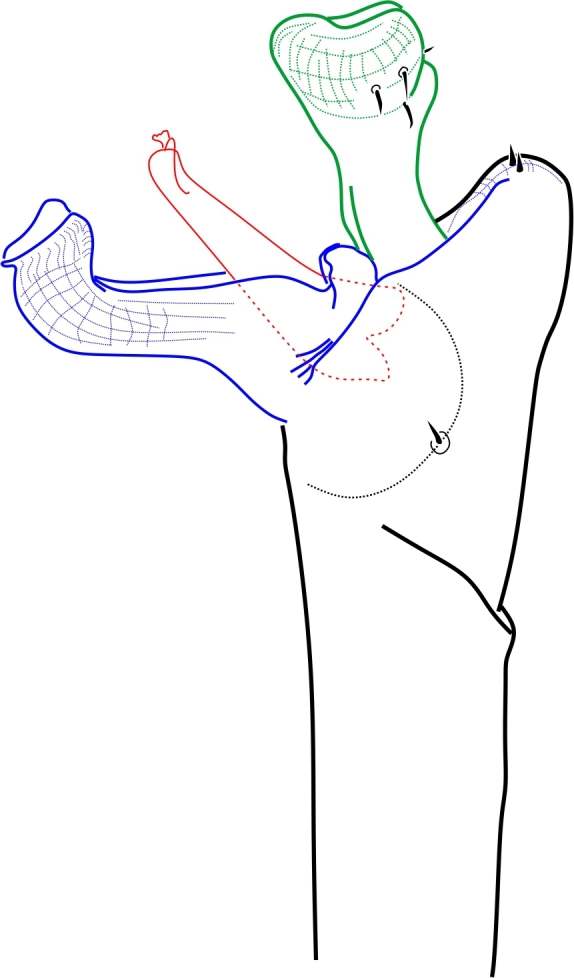
Zalmoxoidea, *Phalangodella* sp., male (MNRJ 2438) from Ecuador. Penis, expanded, lateral view. Drawing by APG/ABK. Blue = stragulum; green = rutrum; red = capsula interna.

**Figure 22.**
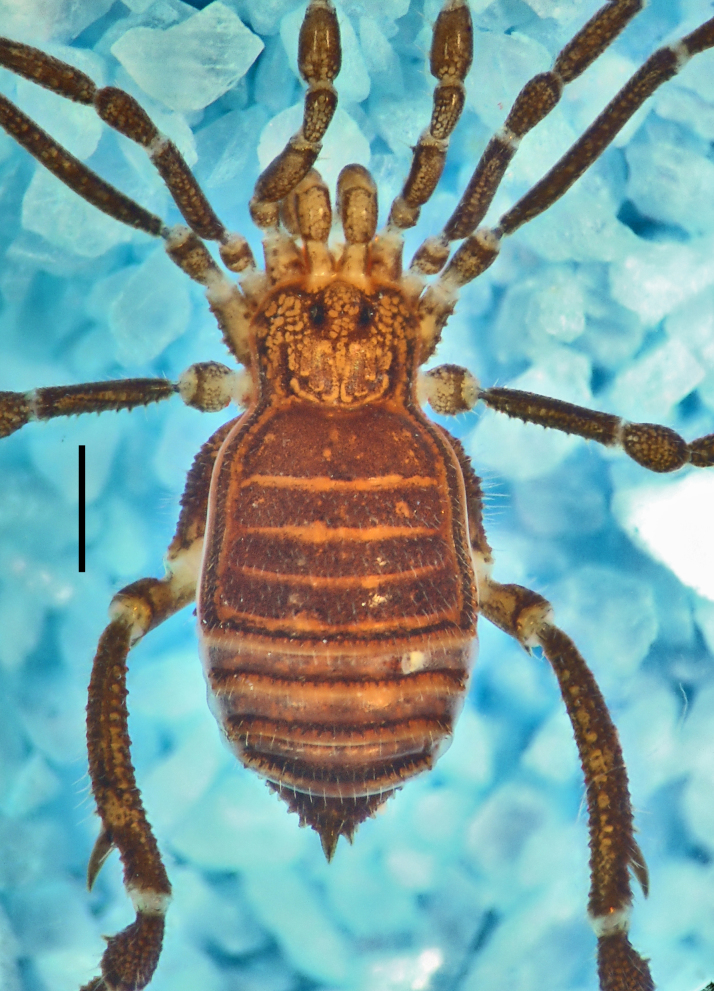
Zalmoxoidea, *Phalangodella* sp., from Manabi, Ecuador, male, habitus, dorsal view (MNRJ 19336). Photo by Daniele R. Souza (with permission). Scale bar = 1 mm.

**Rationale for the placement.** The male genitalia of *Phalangodella* sp. has a jackknife structure exclusive to Zalmoxoidea with a foldable capsula externa (stragulum). The presence of a rudimentary pergula and rutrum justifies its inclusion in Zalmoxoidea, closest to Zalmoxidae and Icaleptidae, although it is not a perfect match with either (Figs 20, 21). The five genera here included in synonymy all share the same body plan (judging by the information available in the literature), and only have been separated in the past due to the oversplitting nature of the Roewerian system. Fig. 22 The extremely narrow marginal ocularium is easily recognizable (*e.g.* Fig. 22), even in the sketch drawings of the previous literature. The revised genus *Phalangodella* is here transferred to Zalmoxidae.

**Included species.**

*Phalangodella aequatorialis* Roewer, 1912 (type species)

*Phalangodella flavipes* (Mello-Leitão, 1949) new comb. for *Langodinus flavipes* Mello-Leitão 1949: 7.

*Phalangodella fulvescens* (Mello-Leitão, 1943) new comb. for *Exlineia fulvescens* Mello-Leitão 1943: 2.

*Phalangodella milagroi* (Mello-Leitão, 1942) new comb. for *Erxlineia milagroi* Mello-Leitão 1942: 315, fig. 1.

*Phalangodella rhinoceros* (Mello-Leitão, 1945) new comb. for *Exlineia rhinoceros* Mello-Leitão 1945: 149, figs. 1–2.

*Phalangodella rugipes* (Roewer, 1949) new comb. for *Cochirapha rugipes* Roewer 1949c: 40, fig. 69.

*Phalangodella urarmata* (Roewer, 1949) new comb. for *Phalpuna urarmata* Roewer 1949c: 41, figs. 71a-e.

**Generic diagnosis.** Dorsal scutum campaniform elongate, densely covered by long-haired setiferous tubercles. Carapace elongate, with ocularium very narrow, marginal as a blunt protuberance. Mesotergum divided into 4 areas by substraight grooves, last groove curved backwards. Both male and female possess femur IV thickened, curved and with distal prolateral spine and tibia IV moderately incrassate. Penis with short scattered macrosetae; rudimentary proto-pergula (projected distal ring) and proto-rutrum (thick mushroom-shaped apical process). Capsula externa jackknife-like unfolding by means of a long stragulum, divided into left and right halves. Capsula interna without lateral sclerites, and with apical expansion as a parastyllar collar. Truncus cylindrical with subdistal fold encircling lateral and ventral parts.
